# Follow-up assessment of patients with Pure Neural Leprosy in a reference center in Rio de Janeiro—Brazil

**DOI:** 10.1371/journal.pntd.0010070

**Published:** 2022-01-11

**Authors:** Izabela Jardim Rodrigues Pitta, Mariana de Andrea Vilas-Boas Hacker, Ligia Rocha Andrade, Clarissa Neves Spitz, Robson Teixeira Vital, Anna Maria Sales, Sergio Luiz Gomes Antunes, Euzenir Nunes Sarno, Marcia Rodrigues Jardim

**Affiliations:** 1 Leprosy Laboratory, Oswaldo Cruz Institute, Fiocruz, Rio de Janeiro, Brazil; 2 Post-Graduate Program in Neurology, Federal University of the State of Rio de Janeiro, Rio de Janeiro, Brazil; 3 Department of Neurology, Pedro Ernesto University Hospital/Rio de Janeiro State University, Rio de Janeiro, Brazil; Hopital Louis Pasteur, FRANCE

## Abstract

**Introduction:**

Pure Neural Leprosy (PNL) is a rare clinical form of leprosy in which patients do not present with the classical skin lesions but have a high burden of the disability associated with the disease. Clinical characteristics and follow up of patients in PNL are still poorly described in the literature.

**Objective:**

This paper aims to describe the clinical, electrophysiological and histopathological characteristics of PNL patients, as well as their evolution after multidrug therapy (MDT).

**Methods:**

Fifty-two PNL patients were selected. Clinical, nerve conduction studies (NCS), histopathological and anti-PGL-1serology were evaluated. Patients were also assessed monthly during the MDT. At the end of the MDT, all of the patients had a new neurological examination and 44 were submitted to another NCS.

**Results:**

Paresthesia was the complaint most frequently reported by patients, and in the neurological examination the most common pattern observed was impairment in sensory and motor examination and a mononeuropathy multiplex. Painful nerve enlargement, a classical symptom of leprosy neuropathy, was observed in a minority of patients and in the motor NCS axonal injuries, alone or in combination with demyelinating features, were the most commonly observed. 88% of the patients did not present any leprosy reaction during MDT. There was no statistically significant difference between the neurological examinations, nor the NCS pattern, performed before and after the MDT.

**Discussion:**

The classical hallmarks of leprosy neuropathy are not always present in PNL making the diagnosis even more challenging. Nerve biopsy is an important tool for PNL diagnosis as it may guide therapeutic decisions. This paper highlights unique characteristics of PNL in the spectrum of leprosy in an attempt to facilitate the diagnosis and management of these patients.

## Author summary

Neuropathy is responsible for most of the disability associated with leprosy disease. The pure neural leprosy (PNL), in spite of being a rare clinical presentation of that, has some singular characteristics that are still poorly studied. In this study, we selected 52 patients at the diagnosis to describe their clinical, neurophysiological and histopathological presentation patterns as well as their evolution during the multidrug therapy (MDT). We confirmed that sensitive symptoms were the most commonly described by PNL patients but neural pain is rare. Also confirmed that mononeuropathy mutiplex was the most common pattern observed at neurological examination and at the nerve conduction studies at the moment of the diagnosis. However we showed that painful nerve enlargement other cardinal signs of leprosy, besides the skin lesions, is not common in this group of patients, There was no statistically significant difference between the neurological examination and NCS performed before and after the MDT.During the MDT88.4% of the patients did not have any leprosy reaction. This may suggest that that MDT was effective in stopping disease progression. Our results highlight the difficulties in the diagnosis of PNL and the progression following MDT in an attempt to prevent further disability in PNL patients.

## Introduction

Leprosy is a long-time known infectious disease caused by *Mycobacterium leprae*, an intracellular pathogen that parasitizes macrophages and Schwann cells [1]. Although it is usually recognized as a skin disease, the neurological complications of leprosy are responsible for most of the disability caused by this disease [2].

Pure neural leprosy (PNL) is described as the presence of clinical evidence of nerve impairment with or without tenderness in the absence of any sign or history of skin lesions. While the classic forms of leprosy with skin lesions have well-described clinical characteristics and follow-up in the medical literature, these aspects in PNL are still poorly studied. PNL is a rare form of the disease, thus its diagnosis and management are a challenge and the clinical evolution of the patients is not well-known [3].

Nerve conduction studies (NCS) are essential to determine the extent of neuropathy and to assess the degree of neural damage in leprosy [4]. This exam is fundamental for PNL patients in order to confirm suspected leprosy neuropathy. In addition, it can help in choosing the best nerve to perform the biopsy necessary for the leprosy diagnosis [3].

This paper aims to describe the clinical, electrophysiological and histopathological characteristics of PNL patients before and after the treatment with multidrug therapy (MDT).

## Methods

### Ethics statement

The study was approved by the Research Ethics Committee of the institution (4.503586) and since the data were obtained from a database there was no need for a written consent.

### Patients

The patients in our study were selected from the database of the Souza Araujo Outpatient Clinic, at the Oswaldo Cruz Institute, a reference center for leprosy in Rio de Janeiro, Brazil. Between 1998 and 2016, 2225 patients were diagnosed with leprosy in this center. Of these, 175 (7.8%) were diagnosed with PNL. There were 71 patients who had neurological examinations that were executed at least one month prior to and two months after the end of MDT. Thirteen patients with comorbidities related to peripheral neuropathies were excluded from the analysis. Six patients were excluded because they did not have the NCS before the start of MDT in our database. Thus, 52 patients that had neurological examination at the moment of diagnosis and at the end of the MDT, as well as NCS before the start of MDT were selected for this study.

### Methods

At moment of diagnosis, all patients were examined by dermatologists to exclude the presence of skin lesions and were submitted to a slit-skin smear analysis. At the neurological examination, all of patients had their tactile, pain, and thermal sensitivity, as well as strength evaluated in the face and in all four limbs. The neurological evaluation was done following the procedures detailed in Vital et al. [5].

All patients were submitted to NCS. The sensory NCS (sNCS) and motor NCS (mNC) were performed in both upper and lower extremities. The methodology and reference values used in our laboratory are the ones described in Vital et al. [5].

All patients were submitted to a sensory nerve biopsy. The biopsied nerve was chosen according to its clinical and neurophysiological impairment. Nerve segments were divided into fragments for routine histopathological studies and to identify the *M*.*leprae* DNA by the polymerase chain reaction (PCR). The methodology and criteria used for the histopathological diagnostics were those described by Antunes et al [6]. The PCR for *M*. *leprae* DNA and the detection of antibodies against phenolic glycolipid-I (PGL-1) were done as described in Jardim et al. [5,7].

For treatment purposes, the patients were classified as either paucibacillary (PB) or multibacillary (MB) depending on the presence of bacilli in the slit-skin smears until 2005. After that, our reference center began to classify PNL patients as MB when acid-fast bacilli (AFB) were detected in the nerve biopsy.

During the MDT, the patients visited the clinic monthly, during which they would take their supervised dose. They were all referred to the neurology service when they had any new neural symptoms or leprosy reactions. At the end of the MDT, the patients were once again examined by a neurologist and 44 of them were submitted to a new NCS. The patients were instructed to return immediately in the case of any neurological deficit or skin lesions during and after the MDT.

Statistical analysis was performed by the McNemar’s and Wilcoxon tests, for respectively categorical and continuous variables. Significance level of 5% was adopted. *Statistical Package for the Social Sciences* (SPSS) v16.0 for Windows was used.

## Results

Fifty-two PNL patients were selected for this study. The mean age of the patients was 47.9 years and the median was 46.5 years; 73.1% (38 patients) were male and 26.9% (14 patients) female. The mean duration of symptoms was 38.2 months prior to diagnosis, (ranging from 2–240 months), while the median was 18 months. The patients had already visited a mean of 3 other services, with a maximum of 5 other services visited, until they were referred to our reference center. The slit-skin smear was negative in all 52 patients.

### Diagnosis

The chief complaints and the neurological examination of the patients at the diagnosis are summarized in Table 1.

**Table 1:** Chief complaints and neurological examination at the diagnosis.

			N	%
Chief Complaints	Positive sensitive symptoms	Neuropathic pain	6	11.5
		Paresthesia	19	36.5
	Negative sensitive symptoms	Hypoesthesia	5	9.6
	Motor symptoms	Weakness	12	23.1
		Muscle atrophy	6	11.5
	Trophic injuries		4	7.7
Neurological Examination	Only impairment in thermal and pain sensation (small fibers)		6	11.5
	Impairment in tactile, pain, and thermal sensations		10	19.2
	Impairment in sensitive and motor examination		31	59.6
	Only impairment in motor examination		5	9.6
	Nerve thickening		33	63.4
Neurological Examination Extension Pattern	Mononeuropathy		16	30.8
	Mononeuropathy multiplex		31	59.6
	Polyneuropathy		5	9.6

**Fig 1:**
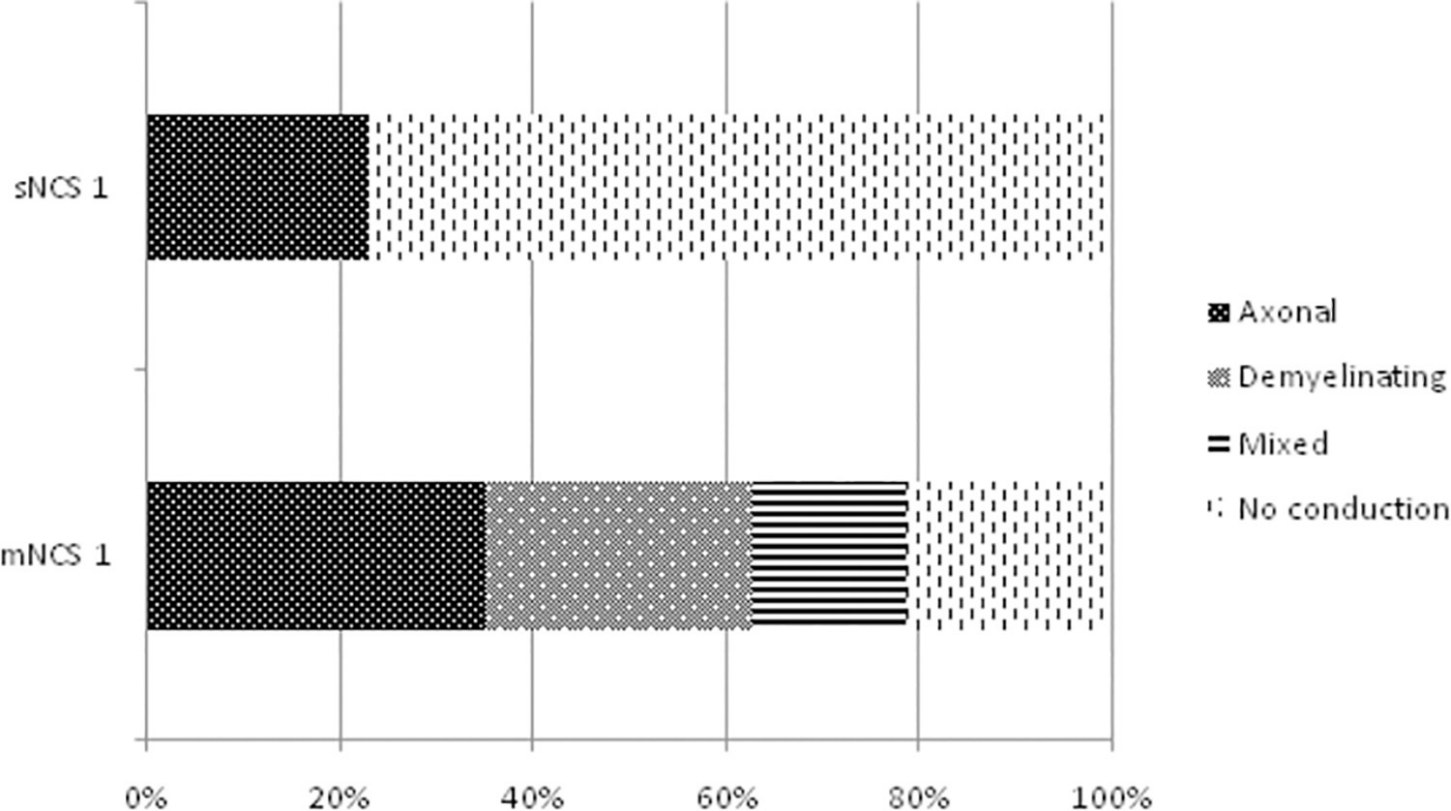
Nerve conduction study (NSC)patterns of the abnormal nerves before the MDT. Mixed: axonal and demyelinating. sNCS: sensory NCS. mNCS: motor NCS.

All of the patients were submitted to NCS before the MDT. 69.2% of the patients (36 patients) presented with a mononeuropathy multiplex, 19.2% (10 patients) with a mononeuropathy, 9.6% (5 patients) with a polyneuropathy and 1.9% (1 patient) had a normal NCS. The NCS patterns of the affected nerves are summarized in Fig 1.

The dorsal cutaneous branch of the ulnar nerve was the most commonly biopsied nerve in our sample (51.9%,27 patients), followed by the sural nerve (32.7%, 17 patients), the superficial peroneal nerve (13.5%, 7 patients), and the digital branch of the median nerve (1.9%, 1 patient).

The PCR detection of *M*.*leprae* DNA was done in 41 of the patients of the sample and the results were positive in 28.8% (15 patients).The detection of anti-PGL-1 antibodies was carried out for 33 patients and of these, the results were positive in 36.5% (19 patients).

Results of the diagnostic tests that were used to confirm PNL carried out for our patient sample are summarized in Table 2.

**Table 2:** Diagnostic tests carried out for the sample of patients.

Diagnostic tests		N
Histopathology	Epithelioid granuloma (“BT”)	8
	AFB + epithelioid granuloma (“BB”)	4
	AFB + mononuclear infiltrate (“BL”)	2
	Mononuclear infiltrate	12
	Mononuclear infiltrate + fibrosis	10
	Fibrosis	1
PCR		10
Anti-PGL-1		5

BT: borderline-tuberculoid. BB: borderline-borderline. BL: borderline-lepromatous. PCR: polymerase chain reaction.

The World Health Organization (WHO) operational classification based on the slit-skin smears classifies all of the patients in this sample as PB, as they all had a negative result. The operational classification based on the number of affected nerves, as used by the Brazilian Ministry of Health, and the presence of AFB in the nerve biopsy, as used at the reference center, is described in Table 3.

**Table 3:** Operational classification of pure neural leprosy based on the number of affected nerves and the presence of acid-fast bacilli (AFB) in the nerve biopsy.

		Classification by the presence of AFB		Total
		PB	MB	
Classification by number of affected nerves	PB	26.9% (14)	3.8% (2)	30.8% (16)
	MB	61.5% (32)	7.7% (4)	69.2% (36)
Total		88.5% (46)	11.5% (6)	100% (52%)

AFB: acid-fast bacilli. PB: paucibacillary. MB: multibacillary.

With the criteria used in the reference center at the time the patients were diagnosed, 98.1% (51 patients) of the sample patients were treated with PB-MDT and 1.9% (1 patient) were treated with MB-MDT.

### Evolution

During the MDT only 5.8% (3 patients) had an isolated neuritis, 3.8% (2 patients) had a type 1 leprosy reaction and 1.9% (1 patient) had a type1 leprosy reaction associated with neuritis. 88.4% (46 patients) did not have any leprosy reaction.

At the end of the MDT, all patients were submitted to a neurological examination. There was no statistically significant change in the number of affected nerves, number of thickened nerves, or the presence of weakness in the neurological examination performed before and after the MDT (p>0.05, Wilcoxon and McNemar’s tests).

Forty-four patients were submitted to NCS after the MDT. At the post-MDT NCS, there was an increase in the number of affected sensitive nerves (mean 2.9vs.3.02, p = 0.048, Wilcoxon). There was no statistically significant difference in the number of affected motor nerves, the presence of conduction block, or the NCS extension or pattern in the NCS performed before and after the MDT.

**Fig 2:**
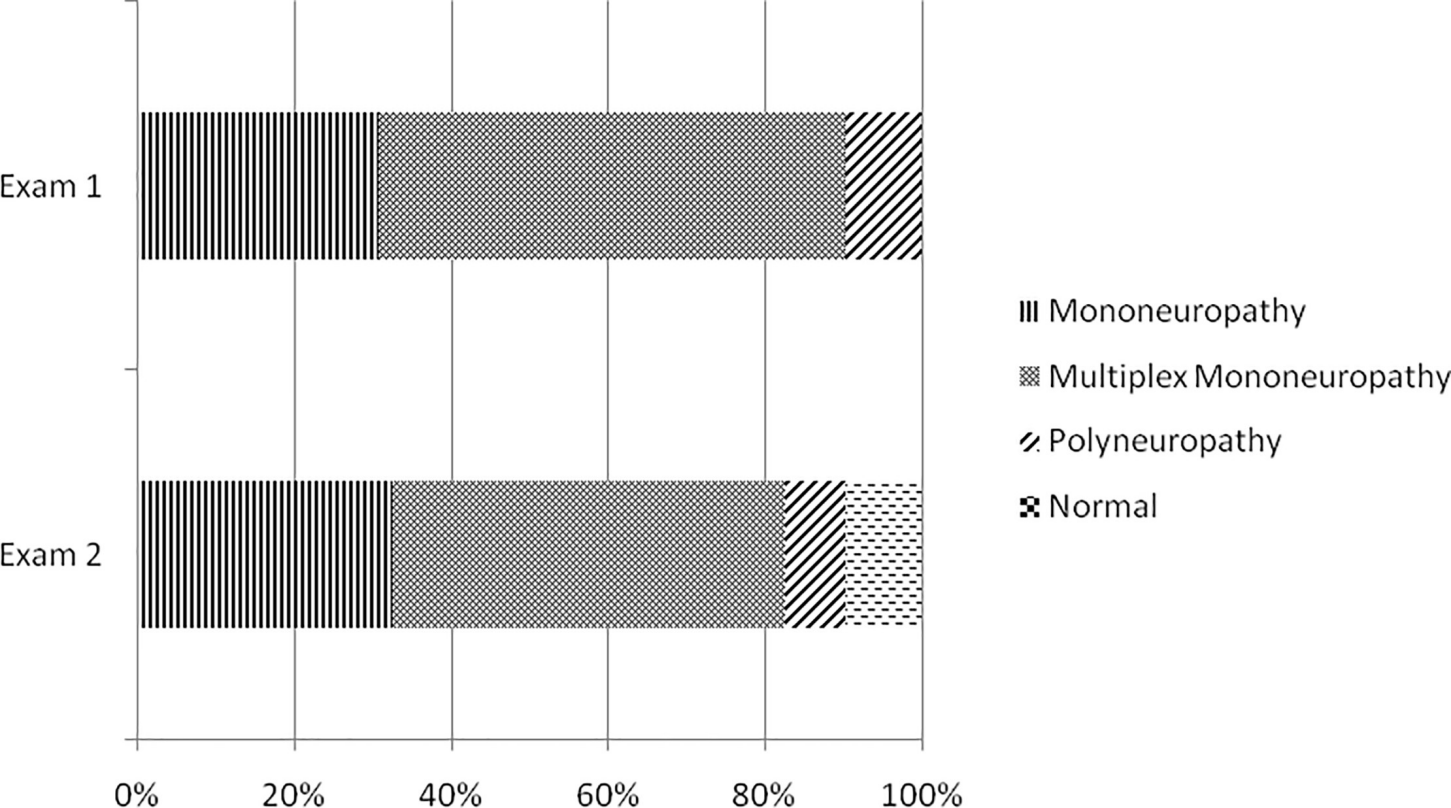
Neurological examination performed before and after the MDT. Exam 1: neurological examination before the MDT. Exam 2: neurological examination after the MDT.

**Fig 3:**
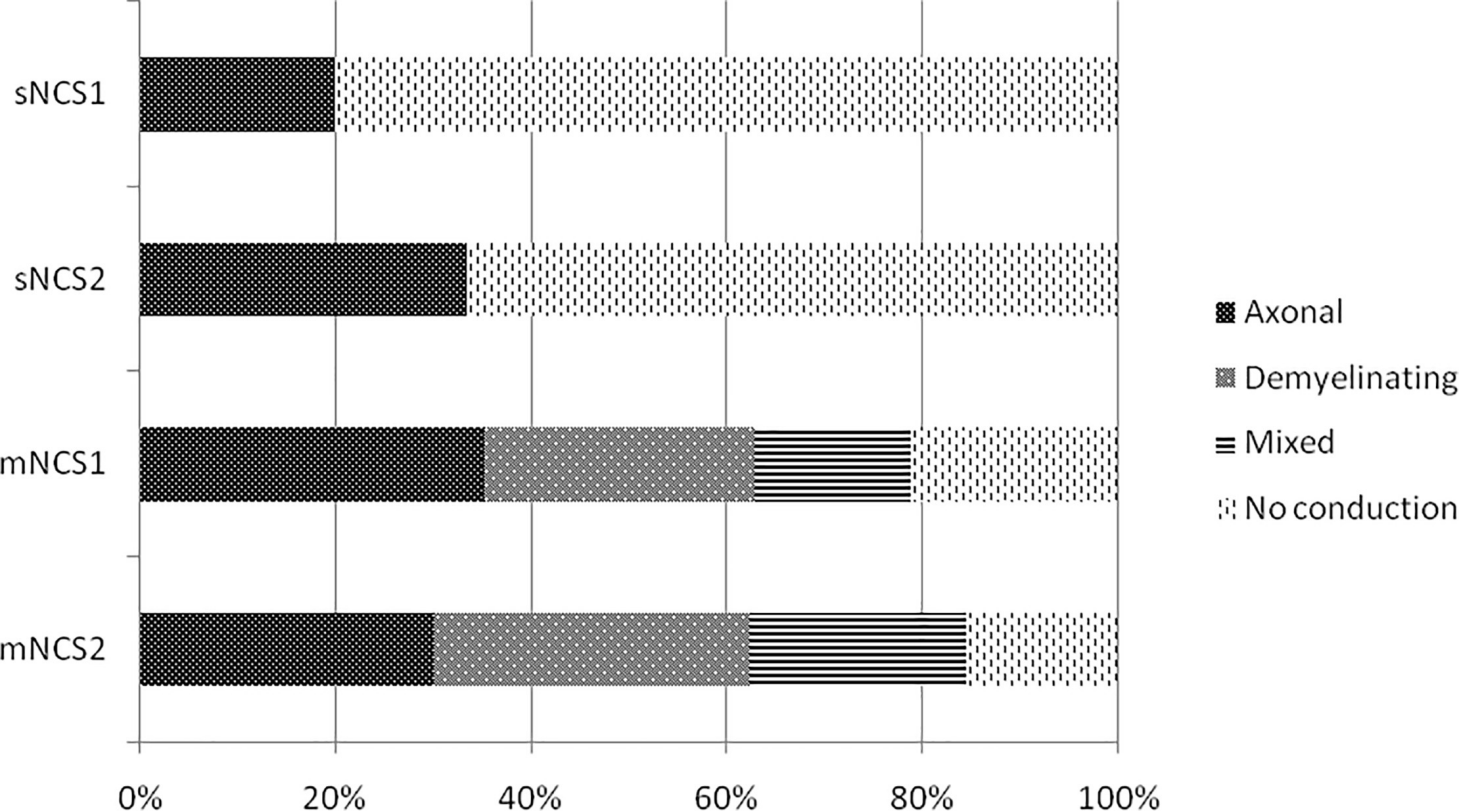
Nerve conduction studies (NCS) performed before and after the MDT. NCS1: NCS performed before the MDT. NCS2: NCS performed after the MDT. sNCS: sensory NCS. mNCS: motor NCS.

The results of the neurological and NCS examinations performed before and after the MDT are summarized in Figs 2 and 3.

## Discussion

The classical forms of leprosy comprise two poles of the disease, the tuberculoid and the lepromatous, as well as the borderline aspect [8]. PNL is a clinical form of the disease in which there are no skin lesions, and thus it is not included in this classification. Over the years some studies have described the presentation and main laboratory test results for PNL patients, but PNL diagnosis is still a challenge and the data available about this form of the disease remain scarce [9]. Regardless of clinical presentation, the sensory and motor losses in leprosy neuropathy are responsible for leprosy disability and the subsequent social consequences, therefore, enhancing the understanding and dispersing the knowledge of this disease will improve the disease management [1,4].

Neuritis is a classical sign of leprosy neuropathy, and is described as neural pain that gets worse with palpation associated to sensory and/or motor impairment [3]. In our sample none of the patients had these clinical hallmarks and most of them did not have any neuropathic pain at the diagnosis or prior to it. This could explain the fact that most of these patients had already visited multiple services before having the leprosy diagnosis, given that without the classical hallmarks the diagnosis is even more difficult. Despite this, in our sample the majority of patients had nerve thickening, which is thought to besecondary to inflammatory response and fibroblast proliferation andisanother classical feature of leprosy neuropathy [10].

The clinical presentation of leprosy neuropathy is directly related to the cellular immune response of the patient [1,4]. However, the asymmetric presentation pattern characterized by the mononeuropathy multiplex is the most commonly described in leprosy neuropathy [11]. In spite of that, some of our patients presented with a symmetric pattern, which can be assumed to be mononeuropathy multiplex that, over time, became confluent, presenting as a polyneuropathy [4]. In our PNL patient sample, there was a large variation in clinical presentation; some patients remained with only a mononeuropathy, despite the long period since the beginning of symptoms, while others had a larger number of affected nerves. This suggests that individual factors may also play a role in the clinical extension of PNL, as occurs in the forms with skin lesions.

It was previously described that the demyelinating patterns are more common in the beginning of leprosy neuropathy, before MDT [12], as seen in our sample, but the presence of axonal loss is widely described in the literature as sign of chronic leprosy neuropathy [3,5]. Although demyelination was present in some of the patients, many of them already had signs of axonal degeneration alone or in combination with demyelinating features in their mNCS, which would suggest that their diagnosis was carried out late in the course of the disease. The long period between the beginning of the symptoms and the diagnosis may corroborate this [12].

Nowadays the nerve biopsy has very few indications, nevertheless, PNL is still one of the few indications of it, as the nerve biopsy is the gold standard for PNL diagnosis and its sensitivity can be increased by PCR testing [13]. The presence of AFB in the biopsied nerve is one of the hallmarks in leprosy histopathology and makes the diagnosis of PNL almost unequivocal, but they are only present in a small percentage of the PNL patients [6]. Our sample also had few patients with the presence of AFB in the biopsied nerve, and this could be explained by the fact that the biopsy only evaluates a small fragment of the affected nerve. However, it could also be an indication that most of the PNL patients are closer to the PB pole of the disease. PCR testing is another way of demonstrating the presence of *M*. *leprae*, and the detection of anti-PGL-1 antibodies enhances the probability of the diagnostic as it represents a higher relative risk of disease, but both of these techniques are only available in a few leprosy centers [7,11].

The WHO currently classifies leprosy into PB and MB based on the number of skin lesion for treatment purposes, but this classification does not specify a separate guideline for PNL [14]. Shukha et al. already proposed that the therapeutic decisions in PNL should be guided by the findings of the nerve biopsy, thus avoiding potential overtreatment with MB-MDT or undertreatment with PB-MDT [14]. The majority of our patients were treated with a PB-MDT, based on the slit-skin smear negativity. Since 88.4% of the patients did not have any leprosy reactions or neuritis during the MDT, our findings may also suggest that the clinical evaluation alone is not enough to guide the decision between PB-MDT and MB-MDT, and the nerve biopsy findings should also be considered.

Some authors hypothesize that PNL could be an initial form of the disease, before the skin lesions. Kaur et al. (1991) described that 30% of PNL patients without MDT treatment presented skin lesions at follow-up [15]. In our sample, all of the patients were treated with MDT, which is a confounding factor, however, the long symptom duration prior to the diagnosis without developing any skin lesion could contradict this idea. As well as in other clinical forms of leprosy, we suggest that in PNL some individual immunological factors could contribute to the development of the disease, making it confined to the nerve [16].

The clinical and neurophysiological evaluation of patients showed no significant difference before and after the MDT. Despite the increase in number of sensitive affected nerves in the NCS after the MDT, it does not imply that it was ineffective. MDT is considered a very effective treatment for leprosy and when a full course is taken properly, relapse is rare [17]. Most of the patients did not have any leprosy reaction or neuritis after the MDT. Therefore, we could assume that the MDT was effective in stopping the infection progression although it was not capable of reverting the damage that was already present.

PNL is still a very common cause of neuropathy in many countries, especially in Latin America and Asia, but is still poorly recognized and understood. Limitations are present at this study, specially that after the end of the MDT the patients were instructed to return to the outpatient clinic in case of new symptoms but it depends on the patient collaboration. This paper aims to highlight the presentation patterns of PNL as well as the progression following MDT in an attempt to facilitate diagnosis and management of the patients.
